# Oxidation of squalene by singlet oxygen and free radicals results in different compositions of squalene monohydroperoxide isomers

**DOI:** 10.1038/s41598-018-27455-5

**Published:** 2018-06-14

**Authors:** Naoki Shimizu, Junya Ito, Shunji Kato, Yurika Otoki, Masashi Goto, Takahiro Eitsuka, Teruo Miyazawa, Kiyotaka Nakagawa

**Affiliations:** 1 Food and Biodynamic Chemistry Laboratory, Graduate School of Agricultural Science, Tohoku University, Sendai, Miyagi 980-0845 Japan; 2 Department of Cell Biology, Division of Host Defense Mechanism, Tokai University School of Medicine, Isehara, Kanagawa 259-1193 Japan; 3 R&D Department, Sunstar Inc., Takatsuki, Osaka, 569-1195 Japan; 4 Food and Biotechnology Innovation Project, New Industry Creation Hatchery Center (NICHe), Tohoku University, Sendai, Miyagi 980-8579 Japan; 5 Food and Health Science Research Unit, Graduate School of Agricultural Science, Tohoku University, Sendai, Miyagi 981-8555 Japan

## Abstract

Oxidation of squalene (SQ) causes a decline in the nutritional value of SQ in foods, as well as an accumulation of SQ oxidation products in skin lipids which lead to adverse skin conditions. However, mechanistic insights as to how SQ is oxidized by different oxidation mechanisms have been limited, and thus effective measures towards the prevention of SQ oxidation have not been identified. In this study, we oxidized SQ by either singlet oxygen oxidation or free radical oxidation, and monitored the formation of the six SQ monohydroperoxide (SQOOH) isomers, the primary oxidation products of SQ, at the isomeric level. While singlet oxygen oxidation of SQ resulted in the formation of similar amounts of the six SQOOH isomers, free radical oxidation of SQ mainly formed two types of isomers, 2-OOH-SQ and 3-OOH-SQ. The addition of β-carotene during singlet oxygen oxidation, and the addition of α-tocopherol during free radical oxidation lead to a dose-dependent decrease in the formation of SQOOH isomers. Such results suggest that the analysis of SQOOH at the isomeric level allows for the determination of the cause of SQ oxidation in various samples, and provides a foothold for future studies concerning the prevention of SQ oxidation.

## Introduction

**Figure 1:**
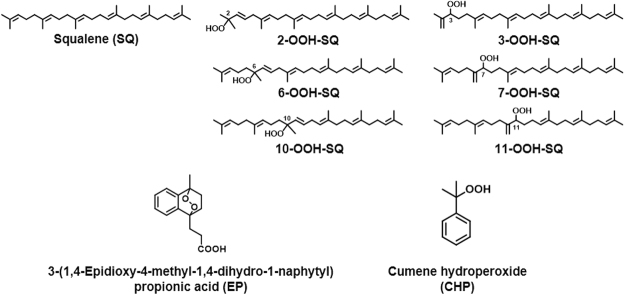
Chemical structures of squalene (SQ), SQ monohydroperoxide (SQOOH) isomers, 3-(1,4-epidioxy-4-methyl-1,4-dihydro-1-naphytyl) propionic acid (EP), and cumene hydroperoxide (CHP).

Squalene (SQ; Fig. 1) is a linear polyunsaturated triterpene that is a key intermediate metabolite during the biosynthesis of cholesterol and other sterols. Shark liver oil, widely applied as a dietary supplement, contains SQ as the principal component, and is the richest natural source of SQ^1^. SQ has also been identified from plant sources including edible oils such as olive oil and amaranth seed oil. Intake of SQ from such dietary sources have been reported to contribute to various beneficial physiological effects. For example, it has been suggested that the dietary administration of SQ suppresses the carcinogenesis of colon cancer^2^, and prevents isoproterenol-induced myocardial infraction^3^ in rats. SQ has also been reported to potentiate the effects of anticancer agents when anticancer agents are emulsified in SQ^4,5^. Such studies point out to the benefits of SQ for the use in nutraceutical and pharmacological purposes as well as its use as a functional food^6,7^.

Interestingly, SQ is also found in rather large amounts in human skin surface lipids, where SQ accounts for about 12% of total lipids^8,9^. Although the function of SQ in skin lipids is not well understood, it has been postulated that SQ plays a role as a lubricant, enabling the movement of lipophilic antioxidants (e.g. vitamin E) on skin, and hence indirectly protects the skin from oxidative stress^10^. Additionally, SQ per se can function as an antioxidant, suggesting the direct involvement of SQ in the protection of the skin^9,11^. While SQ acts as a protective barrier towards the skin, because it occurs on the outermost surface of the human body, SQ is also the target of various oxidative stressors that lead to its oxidation. In fact, SQ is suggested to be the initial target of skin lipid oxidation, forming SQ monohydroperoxide (SQOOH) as the primary oxidation product^12–15^. SQOOH is known to accumulate on human skin, and cause various skin conditions including skin hyperpigmentation^16^, the formation of wrinkles^17^, and the development of inflammatory acne^18^.

Considering the aforementioned beneficial physiological effects of SQ along with the toxicity of SQ oxidation products, the prevention of SQ oxidation is vital to maintain the availability and nutritional value of SQ in foods, as well as to ameliorate the adverse effects of SQOOH towards the skin. A possible way to prevent the oxidation of SQ is the application of antioxidants. However, different types of antioxidants are known to differ in their mechanisms of action; β-carotene (β-Car) is known to be a quencher of singlet oxygen, whereas α-tocopherol (α-Toc) is a quencher of free radicals. Accordingly, the identification of the oxidation mechanism that induces the oxidation of SQ (e.g. singlet oxygen oxidation and free radical oxidation) is necessary in order to select an antioxidant that is effective towards the prevention of SQ oxidation.

One way to evaluate oxidation mechanisms of lipids is to analyze lipid hydroperoxides, the initial oxidation products of lipid oxidation, at the isomeric level^19–24^. This is based on the fact that certain lipids (e.g. linoleic acid) form characteristic lipid hydroperoxide isomers depending on the oxidation mechanism that the lipid was oxidized by^25^. With regard to the oxidation of SQ, only few studies have investigated SQOOH at the isomeric level, and thereby it is unknown whether different oxidation mechanisms yield characteristic SQOOH isomers. Presumably, this is in part due to the lack of an analytical method to investigate SQOOH at the isomeric level. Accordingly, we recently established a method to individually detect the six SQOOH isomers^26^, based on evidence that the use of sodium ions during LC-MS/MS analysis enables the selective analysis of other lipid hydroperoxide isomers^19–24,27,28^.

In this study, to determine whether different oxidation mechanisms yield specific SQOOH isomers, we analyzed *in vitro* oxidation products of SQ which were oxidized by either singlet oxygen oxidation or free radical oxidation. Subsequently, the effect of β-Car and α-Toc on the oxidation of SQ were examined to evaluate whether their different mechanisms of action reflect the effectiveness towards the prevention of SQ oxidation. The results of this study, providing insight into preventative measures towards SQ oxidation, will be valuable in future studies concerning food products containing SQ, as well as dermatological and cosmetic studies that relate to the prevention of skin conditions caused by oxidative stress and lipid oxidation.

## Results

### LC-MS/MS analysis of SQOOH isomers

**Figure 2:**
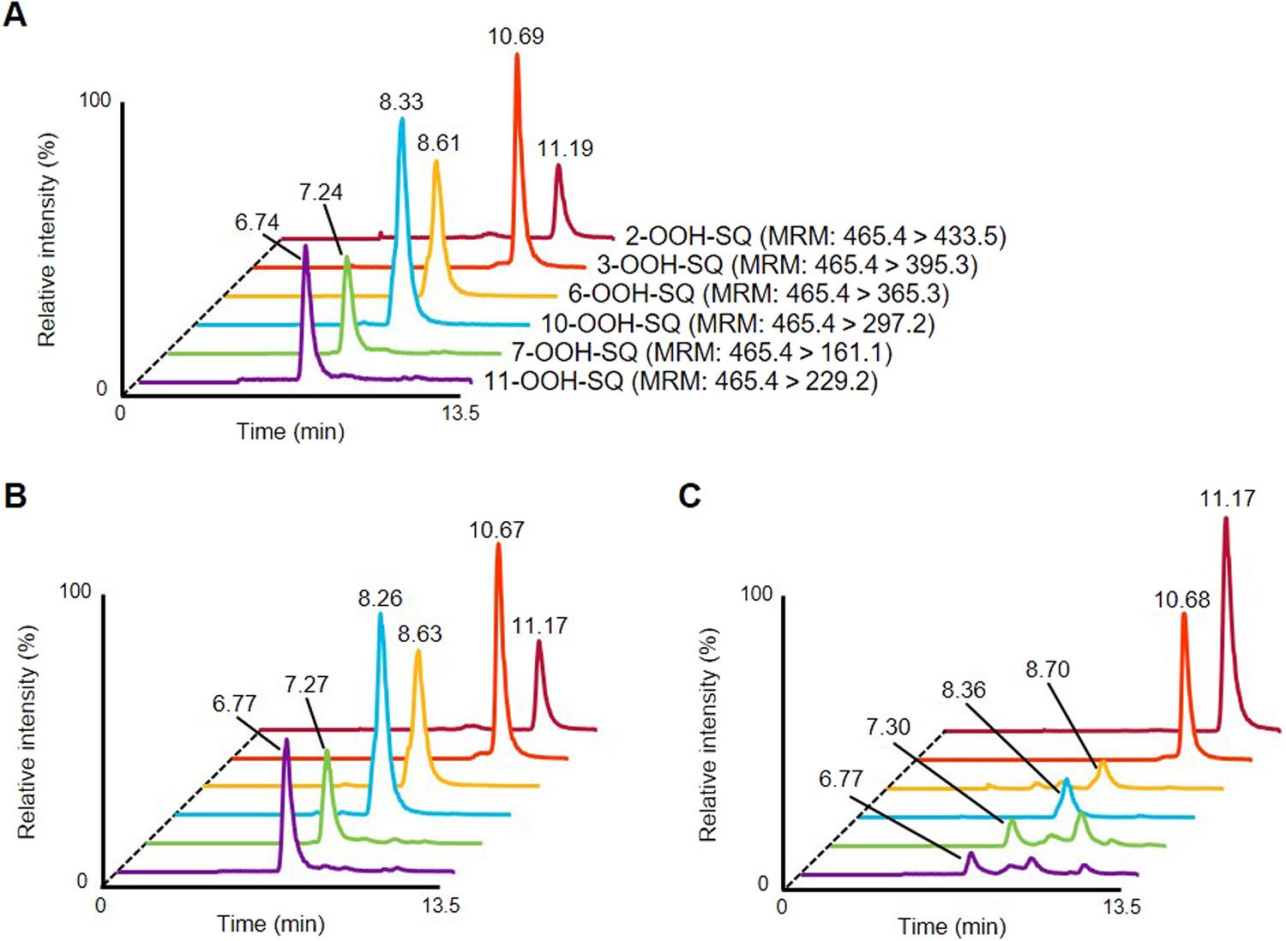
Typical normal phase LC-MS/MS chromatograms analyzing a mixture of 1 ng SQOOH isomer standards (**A**), SQ samples oxidized by singlet oxygen oxidation in the presence of 10 mM EP for 8 hours (**B**), and SQ samples oxidized by free radical oxidation via heating at 50 °C for 8 hours (**C**). Numbers above the peaks represent retention times (min).

Analysis of SQOOH isomers was achieved by adopting our previous normal phase LC-MS/MS method^26^ to a triple-quadrupole mass spectrometer. In accordance with our previous method, MS/MS analysis of the sodium adduct of SQOOH ([SQOOH + Na]^+^) resulted in the production of characteristic fragment ions based on the position of the hydroperoxide group, allowing for the selective detection of SQOOH isomers. A typical chromatogram analyzing a mixture of SQOOH isomer standards is shown in Fig. 2A. Detection limits were in the range of 50–100 pg (0.1–0.2 pmol), achieving around a 100-fold increase in sensitivity compared to our previous method.

### Singlet oxygen oxidation of SQ

**Figure 3:**
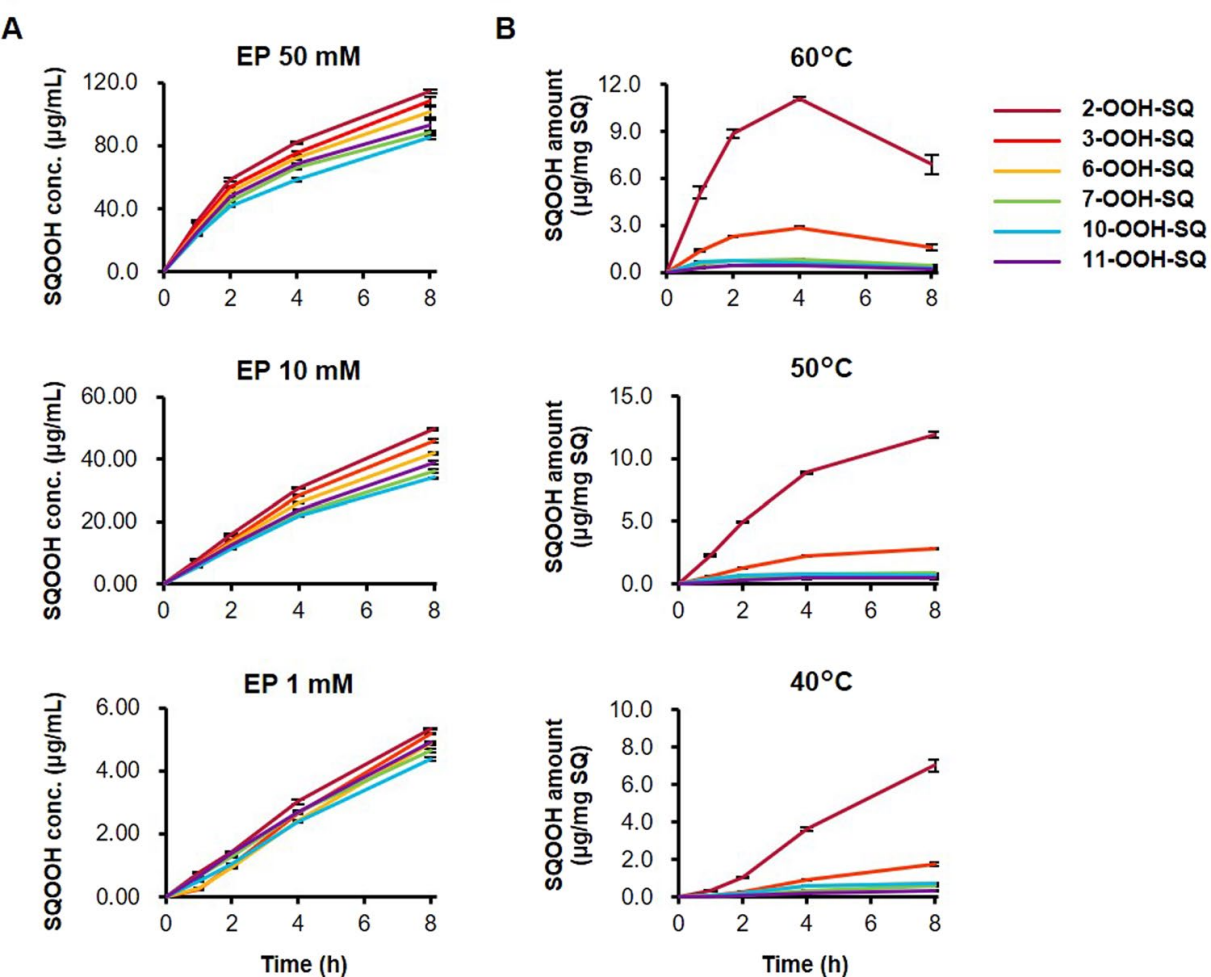
Time-dependent changes in the amount of SQOOH isomers in SQ samples oxidized by singlet oxygen oxidation in the presence of 1, 10, or 50 mM EP (**A**); and samples oxidized by free radical oxidation via heating at 40 °C, 50 °C, or 60 °C (**B**). All samples were prepared in triplicate, and bars denote mean ± SE.

Singlet oxygen oxidation of SQ was investigated with the use of 3-(1,4-epidioxy-4-methyl-1,4-dihydro-1-naphytyl) propionic acid (EP; Fig. 1)^11^, which is known to thermally decompose to yield singlet oxygen. Singlet oxygen oxidation of SQ by EP resulted in the formation of relatively similar amounts of the six SQOOH isomers (Fig. 2B). The amount of SQOOH isomers formed was proportional to the concentration of EP, and SQOOH concentrations tended to increase over time (Fig. 3A). The concentration of total SQOOH in samples oxidized by 1, 10, and 50 mM EP for 8 hours were equivalent to about 3.0, 25.6, and 60.8% of the initial SQ.

**Figure 4:**
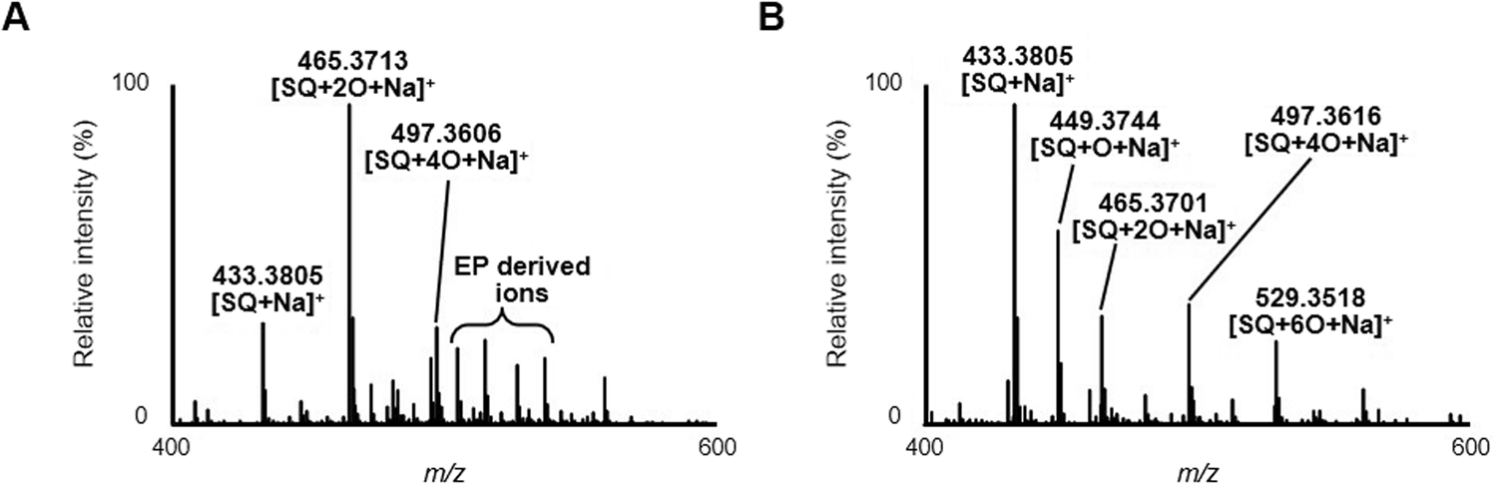
Infusion MS spectra of SQ samples oxidized by either singlet oxygen oxidation in the presence of 10 mM EP for 8 hours (**A**) or samples oxidized by free radical oxidation via heating at 50 °C for 8 hours (**B**).

Because our normal phase LC-MS/MS analysis was specialized to the analysis of the six SQOOH isomers, direct infusion MS analysis of SQ singlet oxygen oxidation products was performed to comprehensively analyze SQ oxidation products (Fig. 4A). As a result, the main peak detected was *m/z* 465.3713 ([SQ + 2 O + Na]^+^, exact mass 465.3709), corresponding to SQOOH. Additionally, *m/z* 497.3606 ([SQ + 4 O + Na]^+^, exact mass 497.3607) which presumably corresponds to SQ dihydroperoxide, was also identified. Other peaks were also observed, but were considered to be derived from EP (e.g. *m/z* 515.1608 corresponding to [2EP + Na]^+^ and *m/z* 537.1443 corresponding to [2EP−H + 2Na]^+^), and other SQ-related peaks except from SQ (*m/z* 433.3805, [SQ + Na]^+^) were not identified.

### Free radical oxidation of SQ

Free radical oxidation of SQ was performed by heating SQ in the presence of cumene hydroperoxide (CHP; Fig. 1) as a radical initiator. The main SQOOH isomer formed by the free radical oxidation of SQ was 2-OOH-SQ, followed by 3-OOH-SQ (Fig. 2C). The other SQOOH isomers (i.e. 6, 7, 10, and 11-OOH-SQ) were also formed, but in lesser amounts. The composition of SQOOH isomers formed after the free radical oxidation of SQ for 8 hours at 50 °C was 2-OOH-SQ, 68.6%; 3-OOH-SQ, 16.2%; 6-OOH-SQ, 3.9%; 7-OOH-SQ, 4.8%; 10-OOH-SQ, 3.8%; 11-OOH-SQ, 4.1%. While the free radical oxidation of SQ at 40 and 50 °C resulted in a time-dependent increase in the amount of SQOOH isomers formed, the free radical oxidation of SQ at 60 °C induced the decomposition of SQOOH after 4 hours of heating (Fig. 3B). The concentration of total SQOOH in samples oxidized at 40, 50, and 60 °C for 8 hours were equivalent to about 1.12, 1.74, and 0.97% of the initial SQ, respectively.

Infusion MS analysis of the free radical oxidation products of SQ identified SQ oxidation products other from SQOOH (Fig. 4B). Aside from SQ (*m/z* 433.3805, [SQ + Na]^+^), the largest peak observed was *m/z* 449.3744 ([SQ + O + Na]^+^, exact mass 449.3759) which may correspond to either SQ epoxide or SQ hydroxide. Another large peak observed was *m/z* 465.3701 ([SQ + 2 O + Na]^+^, exact mass 465.3709) corresponding to SQOOH. However, considering that SQ epoxide and SQ hydroxide was formed, this peak was presumably a mixture of SQ oxidation products including SQOOH, SQ diepoxide, and SQ dihydroxide. Further SQ oxidation products such as *m/z* 497.3616 ([SQ + 4 O + Na]^+^, exact mass 497.3607) and *m/z* 529.3518 ([SQ + 6 O + Na]^+^, exact mass 529.3505) were also observed.

### Inhibition of the singlet oxygen oxidation of SQ by β-Car and α-Toc

**Figure 5:**
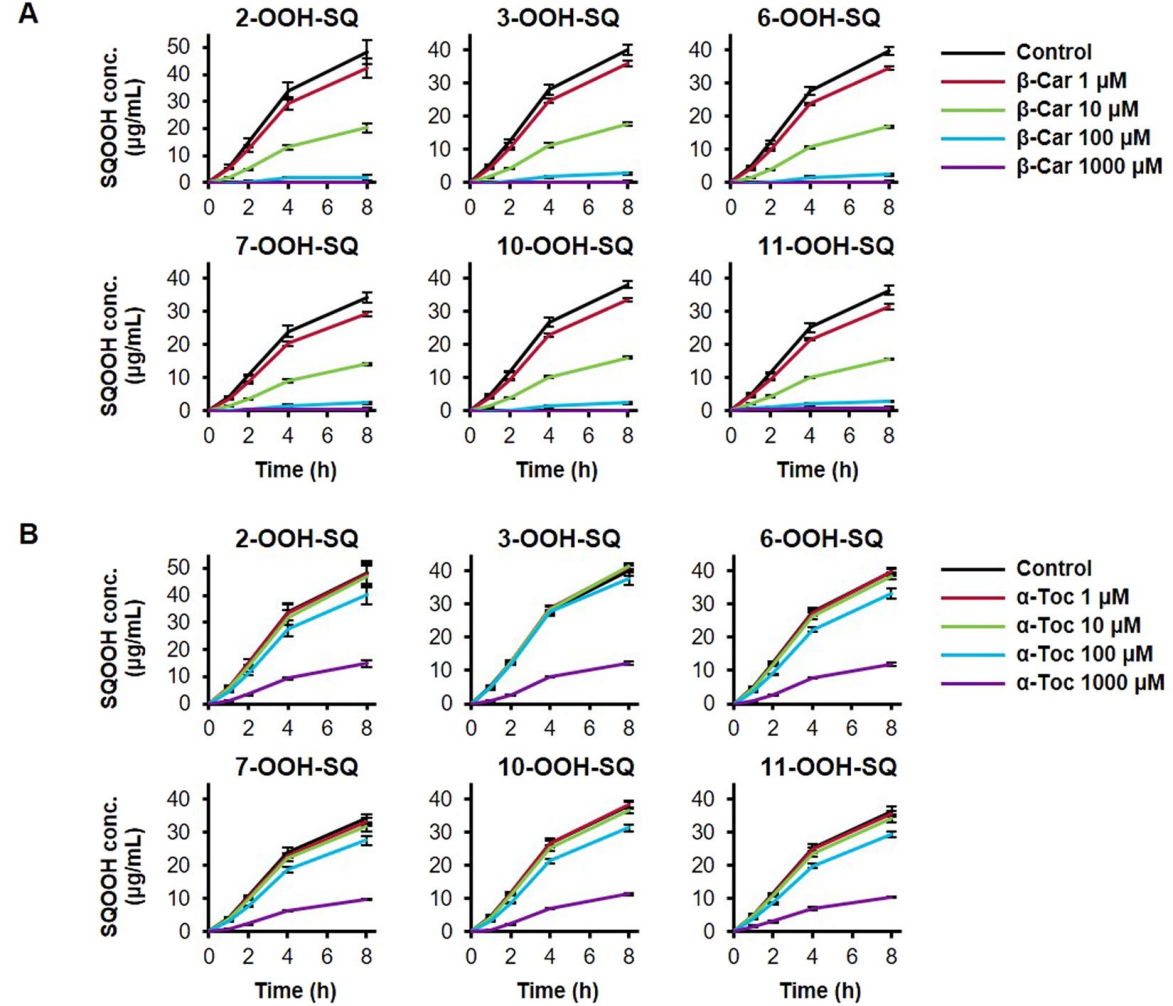
Time-dependent changes in the amount of SQOOH isomers in SQ samples oxidized by singlet oxygen oxidation in the presence of 10 mM EP and 0 (control), 1, 10, 100, or 1,000 μM either β-Car (**A**) or α-Toc (**B**). All samples were prepared in triplicate, and bars denote mean ± SE.

The singlet oxygen oxidation of SQ was dose-dependently inhibited by β-Car in the range of 1–1,000 μM per 1 mg/mL SQ (Fig. 5A). Notably, the addition of 10 μM β-Car approximately reduced the formation of SQOOH by 60%. The effect of β-Car on the formation of SQOOH demonstrated no differences between the six isomers; all six SQOOH isomers were formed in equal amounts regardless of the dose of β-Car. α-Toc also dose-dependently inhibited the oxidation of SQ, but to a lower extent compared with β-Car (Fig. 5B). The addition of 1000 μM α-Toc approximately reduced the formation of SQOOH by 70%. Similar to β-Car, the effect of α-Toc on SQOOH formation demonstrated no differences between the six SQOOH isomers.

### Inhibition of the free radical oxidation of SQ by β-Car and α-Toc

**Figure 6:**
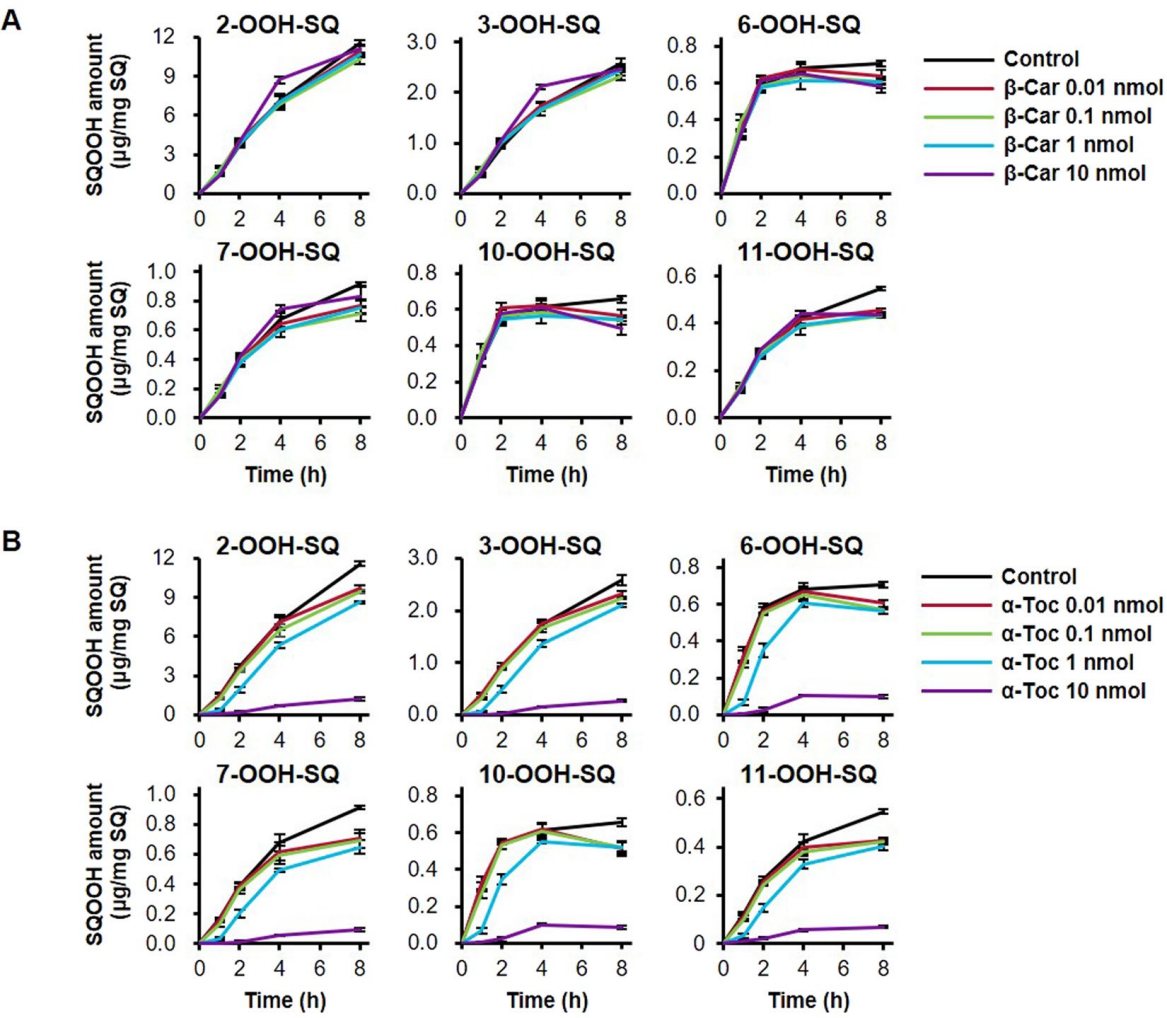
Time-dependent changes in the amount of SQOOH isomers in SQ samples oxidized by free radical oxidation via heating at 50 °C in the presence of 0 (control), 0.01, 0.1, 1, or 10 nmol either β-Car (**A**) or α-Toc (**B**). All samples were prepared in triplicate, and bars denote mean ± SE.

In the range of 0.01–10 nmol per 1 mg SQ, β-Car did not demonstrate much effect on the free radical oxidation of SQ (Fig. 6A), whereas α-Toc inhibited the formation of all six SQOOH isomers (Fig. 6B). The strength of the inhibitory effects of α-Toc demonstrated similar trends in the range of 0.01–1 nmol, where the formation of SQOOH was reduced by about 15%. On the other hand, the addition of 10 nmol α-Toc resulted in a large decrease in the formation of SQOOH isomers, and reduced the formation of SQOOH by about 85%.

## Discussion

SQ is known to possess various beneficial physiological effects as well as antioxidative properties on human skin^2–11^. The oxidation of SQ not only declines the biological availability of SQ, but also its primary oxidation product, SQOOH, possesses adverse effects towards the human skin^16–18^. However, mechanistic insights as to how SQ is oxidized to SQOOH have not been thoroughly investigated to date, and hence studies on measures to prevent the oxidation of SQ have been limited. Studies on how SQ is oxidized by different oxidation mechanisms should provide a foothold for future studies concerning the prevention of SQ oxidation.

Therefore, in this study, we investigated how SQ is oxidized to SQOOH by two oxidation mechanisms, singlet oxygen oxidation and free radical oxidation. Singlet oxygen oxidation of SQ resulted in the formation of SQOOH as the main oxidation product (Fig. 4A). With the use of our normal phase LC-MS/MS method that enables the individual analysis of the six SQOOH isomers^26^, it was identified that all six SQOOH isomers were formed in similar amounts (Fig. 2B). While we and others have previously reported that singlet oxygen oxidation of SQ results in the formation of six types of SQOOH isomers^14,15^, we believe this is the first study that reveals the fact that the six isomers are formed in nearly equal amounts. On the other hand, free radical oxidation of SQ produced a variety of SQ oxidation products, which presumably included SQ epoxides, SQ hydroxides, and other highly oxidized products as well as SQOOH (Fig. 4B). Although the free radical oxidation of SQ has been investigated previously, such studies have suggested the formation of mainly hydroxides, epoxides, and cyclic dihydroperoxides^29–31^, and to the best of our knowledge, the formation of SQOOH by the free radical oxidation of SQ has not been reported previously. Interestingly, the main SQOOH isomers formed by the free radical oxidation of SQ were 2-OOH-SQ and 3-OOH-SQ (Fig. 2C), contrary to singlet oxygen oxidation where all six isomers were formed in nearly equal amounts. This formation of 2-OOH-SQ and 3-OOH-SQ by free radical oxidation was also observed by using different radical initiators such as 2,2′-azobis (4-methoxy-2,4-dimethylvaleronitrile) (MeO-AMVN) and hydroxyl radicals as well (Supplementary Figs S1 and S2). Such results strongly suggest that the analysis of SQOOH isomers can be applied to determine the oxidation mechanism that causes the oxidation of SQ in various samples including SQ containing foods (e.g. olive oil) and skin lipids.

**Figure 7:**
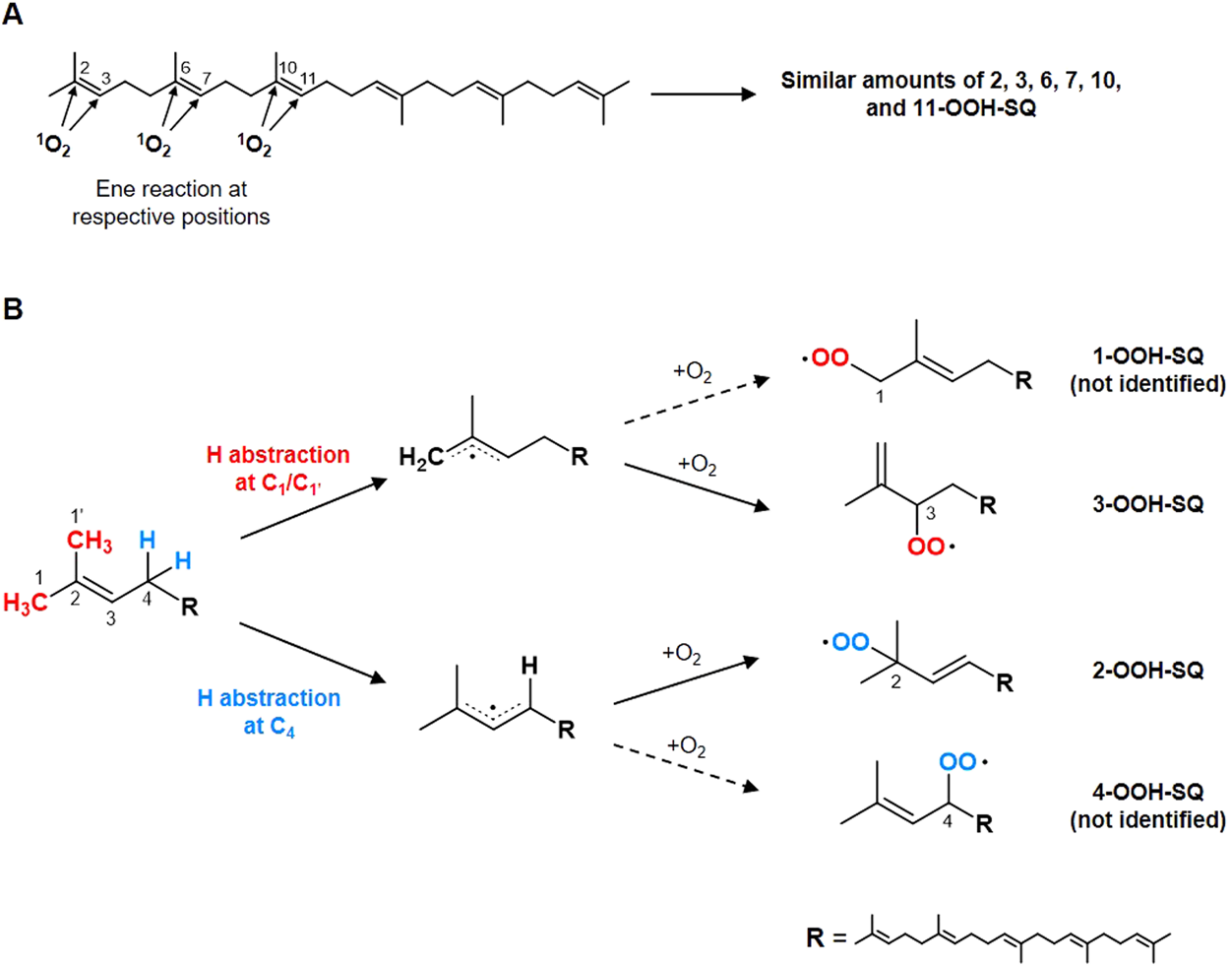
Predicted pathways of the singlet oxygen oxidation of SQ (**A**) and free radical oxidation of SQ (**B**).

The reason as to why singlet oxygen oxidation of SQ produces SQOOH isomers in nearly equal amounts may be explained by the mechanism of singlet oxygen oxidation. During singlet oxygen oxidation, singlet oxygen acts as an electrophilic reagent that reacts with double bonds via the ene reaction to form hydroperoxides^25,32^. Therefore, the main force that initiates the singlet oxygen oxidation of SQ is the electrostatic interaction between singlet oxygen and the double bond of SQ. Thus, it can be assumed that the position of the double bond has minimal effect on where the attacks by singlet oxygen are made, and leads to the formation of equal amounts of SQOOH isomers (Fig. 7A).

On the other hand, why the free radical oxidation of SQ produces mainly 2 and 3-OOH-SQ may be more complicated. Free radical oxidation of lipids initiates via the formation of a lipid radical, which typically occurs through the abstraction of a hydrogen atom from a C–H bond^25,33^. Thereby, the position where the hydrogen abstraction takes place affects the position of the hydroperoxide group in free radical oxidation. While mechanistic studies on the free radical oxidation of SQ have not been conducted, in a previous study, Pogliani *et al*. investigated the molecular dynamics of SQ in which they concluded by saying that SQ undergoes a conformational equilibrium by which the mobile tails of SQ (i.e. the ends of the SQ chain) move around the rigid central portion, thus sweeping the surrounding space and protecting the central part from reactions^34^. Taking this into account, it can be hypothesized that the hydrogen atoms binding to the ends of the SQ chain are more susceptible to hydrogen abstraction than hydrogen atoms binding to other central positions of SQ. Assuming that hydrogen abstraction occurs mainly at the ends of the SQ chain (i.e. C_1_ and C_4_ positions), the main SQOOH isomers formed by free radical oxidation are presumably 1 and 3-OOH-SQ via hydrogen abstraction at the C_1_ position, and 2 and 4-OOH-SQ via hydrogen abstraction at the C_4_ position. However, 1 and 4-OOH-SQ were not identified in this study. While there are no previous studies examining the SQOOH isomers formed by the free radical oxidation of SQ, free radical oxidation of several fragrance terpenes that share a similar structure with SQ (e.g. linalool and geraniol) have been investigated in detail^35–37^. In one of such studies, Sköld *et al*. proposed that the free radical oxidation of linalool hypothetically produces linalool hydroperoxide isomers bearing hydroperoxide groups at the C_8_, C_7_, C_6_, and C_5_ positions, which positionally correspond to 1, 2, 3, and 4-OOH-SQ, respectively^35^. However, the linalool isomers that were actually detected were linalool-7-OOH and linalool-8-OOH, corresponding to 2 and 3-OOH-SQ^35,37^. Moreover, linalool-7-OOH, corresponding to 2-OOH-SQ, is known to form in larger amounts than linalool-8-OOH, corresponding to 3-OOH-SQ, presumable due to the secondary hydrogen atoms preferably being abstracted compared to primary hydrogen atoms^37^. These studies are consistent with our present findings that the free radical oxidation of SQ mainly produces 2 and 3-OOH-SQ.

We therefore propose that the free radical oxidation of SQ follows the mechanism described in Fig. 7B. The mobility of the ends of the SQ chain mainly induces hydrogen abstraction from the C_1_ and C_4_ positions of SQ. As a result of the hydrogen abstraction from the C_1_ position, peroxyl radicals corresponding to 1 and 3-OOH-SQ may be formed, but considering their stabilities, the secondary peroxyl radical (i.e. 3-OO•-SQ) is more stable than the primary peroxyl radical (i.e. 1-OO•-SQ), thus favoring the formation of 3-OOH-SQ. Similarly, since the peroxyl radical corresponding to 2-OOH-SQ, being a tertiary peroxyl radical, is more stable than the peroxyl radical corresponding to 4-OOH-SQ, a secondary peroxyl radical, hydrogen abstraction at the C_4_ position of SQ prefers the formation of 2-OOH-SQ. As a result of such reactions, free radical oxidation of SQ mainly forms 2 and 3-OOH-SQ.

Since it was identified based on the above findings that the analysis of SQOOH isomers allows for the evaluation of oxidation mechanisms, the effect of β-Car and α-Toc on the oxidation of SQ were examined to evaluate whether their different mechanisms of action reflect the effectiveness towards the prevention of SQ oxidation. As a result, the singlet oxygen oxidation of SQ was dose-dependently inhibited by β-Car, a quencher of singlet oxygen, whereas the free radical oxidation of SQ was inhibited by α-Toc, a quencher of free radicals. The fact that α-Toc is also a quencher of singlet oxygen resulted in the inhibition of the singlet oxygen oxidation of SQ by α-Toc. However, the efficiency of the inhibition of singlet oxygen oxidation by α-Toc was about 100-fold lower than that of β-Car, which agrees with the study by Mukai *et al*. in which they suggested that the singlet oxygen quenching rate of β-Car is around 100-fold higher than that of α-Toc^38^. Such results emphasize that identifying oxidation mechanisms by SQOOH isomer analysis, and then applying an antioxidant that is effective towards that oxidation mechanism is an effective way to prevent the oxidation of SQ.

Although β-Car and α-Toc both inhibited the oxidation of SQ, since both antioxidants were particularly effective towards only one of either singlet oxygen oxidation or free radical oxidation, a further screening of other antioxidants may be necessary. In a previous study, we reported that γ-tocotrienol is effective towards reducing inflammatory responses caused by SQOOH in HaCaT keratinocytes^39^, and Wang *et al*. reported that carnosic acid effectively protects the oxidation of SQ under accelerated oxidation conditions^40^. Examination of the effects of such antioxidants on the oxidation of SQ through the analysis of SQOOH isomers may be of particular interest. Also, adopting our current method and substituting β-Car and α-Toc with other antioxidants may enable the screening of other antioxidants that are effective towards the inhibition of singlet oxygen oxidation and/or free radical oxidation of SQ. Investigation of such may be the target of later studies.

To conclude, in this study, we performed LC-MS/MS analysis of SQ oxidation products oxidized by either singlet oxygen oxidation or free radical oxidation. As a result, it was identified that the composition of SQOOH isomers formed through each oxidation mechanism was different, indicating that the analysis of SQOOH at the isomeric level allows for the determination of the oxidation mechanisms of SQ. We also investigated the effects of β-Car and α-Toc and found that each antioxidant was effective towards singlet oxygen oxidation and free radical oxidation of SQ, respectively. The application of our current study ranges from food samples, where the preservation of SQ is essential to maintain the nutraceutical benefits of SQ, to skin lipids, where SQOOH possesses adverse effects, and provides a foothold for future studies concerning the prevention of SQ oxidation.

## Methods

### Materials

SQ, β-Car, and α-Toc were obtained from Wako Pure Chemical Industries, Ltd. (Osaka, Japan). Since the obtained SQ contained noticeable amounts of SQOOH and about 0.05% vitamin E as a stabilizer, SQ was purified by semi-preparative HPLC prior to use. EP was obtained from Waken Btech Co., Ltd. (Kyoto, Japan). CHP (containing ca. 20% aromatic hydrocarbons) was obtained from Tokyo Chemical Industry Co., Ltd. (Tokyo, Japan). SQOOH isomers (i.e. 2, 3, 6, 7, 10, and 11-OOH-SQ) were prepared from SQ as described previously^26^. The purities of SQOOH isomers were >95% judged based on HPLC-UV analysis. All other reagents were of analytical grade or higher.

### Singlet oxygen oxidation of SQ

In an amber vial, 50 μL of 2.0 mg/mL purified SQ in ethanol/chloroform (1:1, v/v) was mixed with 50 μL of 2, 20, or 100 mM EP in ethanol/chloroform (1:1, v/v). Final concentrations were 1.0 mg/mL SQ and 1, 10, or 50 mM EP. The mixture was incubated at 20 °C for 1, 2, 4, or 8 h. All procedures except for the incubation were conducted at 4 °C to prevent the decomposition of EP. Each sample was prepared in triplicate.

### Free radical oxidation of SQ

In an amber vial, purified SQ (1 mg) was mixed with 10 μL of 1 mg/mL CHP in hexane, and the solution was evaporated under nitrogen stream. Oxygen gas was enclosed in the vial, and the cap of the vial was wrapped with Parafilm M (Bermis Company, Inc.; WI, USA) to prevent oxygen from leaking. The samples were then heated at 40, 50, or 60 °C for 1, 2, 4, or 8 h. Each sample was prepared in triplicate.

### Evaluation of the effects of antioxidants on the oxidation of SQ

For the evaluation of the effects of antioxidants on the singlet oxygen oxidation of SQ, 40 μL of 2.5 mg/mL purified SQ in ethanol/chloroform (1:1, v/v) was mixed with 10 μL of 0, 0.01, 0.1, 1, or 10 mM β-Car or α-Toc in ethanol/chloroform (1:1, v/v) in an amber vial. The solution was further mixed with 50 μL of 20 mM EP in ethanol/chloroform (1:1, v/v), and incubated at 20 °C for 1, 2, 4, or 8 h. Final concentrations were 1.0 mg/mL SQ, 10 mM EP, and 0, 1, 10, 100, or 1,000 μM β-Car or α-Toc.

To evaluate of the effects of antioxidants on the free radical oxidation of SQ, 1 mg of purified SQ was mixed with 0, 0.01, 0.1, 1, or 10 nmol β-Car or α-Toc. The solution was further mixed with 10 μL of 1 mg/mL CHP, and was evaporated under nitrogen stream. Oxygen gas was enclosed in the vial as described above, and the samples were heated at 50 °C for 1, 2, 4, or 8 h. All samples were prepared in triplicate.

### Normal phase LC-MS/MS analysis of SQOOH isomers

LC-MS/MS analysis of SQOOH isomers was conducted according to a previously described method^26^ with modifications. Samples oxidized by singlet oxygen oxidation were diluted 100-fold in hexane, and samples oxidized by free radical oxidation were diluted such that the initial concentration of SQ was 0.1 mg/mL in hexane prior to analysis. The samples (10 μL) were analyzed with a 4000 QTRAP mass spectrometer equipped with an ExionLC HPLC/UHPLC system (SCIEX; Tokyo, Japan) under electrospray ionization (positive). A silica column (Inertsil SIL 100A 5 μm, 2.1 × 250 mm; GL Sciences Inc.; Tokyo, Japan) was eluted with hexane/2-propanol (100:0.5, v/v) as the mobile phase at a flow rate of 0.2 mL/min. Methanol/ethanol (1:1, v/v) containing 0.1 mM sodium acetate at a flow rate of 0.1 mL/min was mixed with the eluate at the post column via a gradient mixer. The MS parameters were as follows: curtain gas, 30.0 psi; collision gas, 7.0 psi; ion spray voltage, 5500.0 V; temperature, 400.0 °C; ion source gas 1, 80.0 psi; ion source gas 2, 60.0 psi. Multiple reaction monitoring (MRM) transitions used for the detection of each SQOOH isomer were as follows: 2-OOH-SQ, 465.4 > 433.5; 3-OOH-SQ, 465.4 > 395.3; 6-OOH-SQ, 465.4 > 365.3; 7-OOH-SQ, 465.4 > 161.1; 10-OOH-SQ, 465.4 > 297.2; 11-OOH-SQ, 465.4 > 229.2. The SQOOH concentration of the samples were determined based on external calibration curves prepared with synthetic SQOOH standards.

### Infusion MS analysis of SQ oxidation products

SQ oxidation products were diluted in methanol such that the initial concentration of SQ was 1 μg/mL. The samples were directly infused into a micrOTOF-Q II mass spectrometer (Bruker Daltonik; Bremen, Germany) under electrospray ionization (positive) at a flow rate of 150 μL/h. The MS parameters were set as follows: end plate offset, 500 V; capillary, 4500 V; nebulizer, 1.6 bar; dry gas, 6.0 L/min; dry temp., 180 °C; funnel 1 RF, 300.0 Vpp; funnel 2 RF, 400.0 Vpp; isCID energy, 0 eV; hexapole RF, 400.0 Vpp; ion energy, 6.0 eV; low mass, 200.0 *m/z*, collision energy, 6.0 eV; collision RF, 250.0 Vpp; transfer time, 32.0 μs; pre pulse storage, 8.0 μs.

### Data availability

The datasets generated during and/or analyzed in this study are available from the corresponding author on reasonable request.

## Electronic supplementary material

Supplementary material
