# Supplementary material for: Oxidation of squalene by singlet oxygen and free radicals results in different compositions of squalene monohydroperoxide isomers
Source: Sci Rep. 2018 Jun 14;8:9116. doi: 10.1038/s41598-018-27455-5 (PMC6002538; doi:10.1038/s41598-018-27455-5)
Supplement: Supplementary file 1 — Supplementary material [file 41598_2018_27455_MOESM1_ESM.pdf]

Supplementary material

Oxidation of squalene by singlet oxygen and free radicals results in different compositions of squalene monohydroperoxide isomers

Naoki Shimizu[1], Junya Ito[1], Shunji Kato[1,2], Yurika Otoki[1], Masashi Goto[3], Takahiro Eitsuka[1], Teruo Miyazawa[4,5], and Kiyotaka Nakagawa[1*]

[1]Food and Biodynamic Chemistry Laboratory, Graduate School of Agricultural Science, Tohoku University, Sendai, Miyagi 980-0845, Japan

[2]Department of Cell Biology, Division of Host Defense Mechanism, Tokai University School of Medicine, Isehara, Kanagawa 259-1193, Japan

[3]R&D Department, Sunstar Inc., Takatsuki, Osaka 569-1195, Japan

[4]Food and Biotechnology Innovation Project, New Industry Creation Hatchery Center (NICHe), Tohoku University, Sendai, Miyagi 980-8579, Japan

[5]Food and Health Science Research Unit, Graduate School of Agricultural Science, Tohoku University, Sendai, Miyagi 981-8555, Japan

*Corresponding author: nkgw@m.tohoku.ac.jp

Supplementary Figures

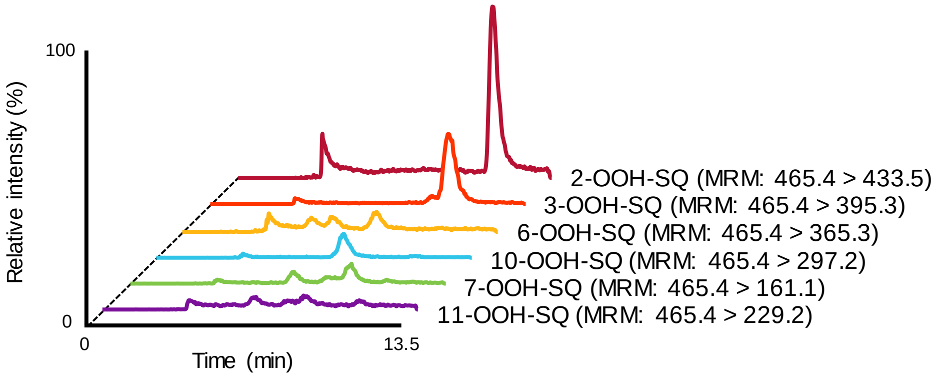

Supplementary Fig. S1. Normal phase LC-MS/MS chromatogram demonstrating the distribution of SQOOH isomers formed by the free radical oxidation of SQ using 2,2'-azobis (4-methoxy-2,4-dimethylvaleronitrile) (MeO-AMVN) as the radical source. In accordance with the free radical oxidation by cumene hydroperoxide, the main SQOOH isomers formed by the oxidation by MeO-AMVN were 2 and 3-OOH-SQ.

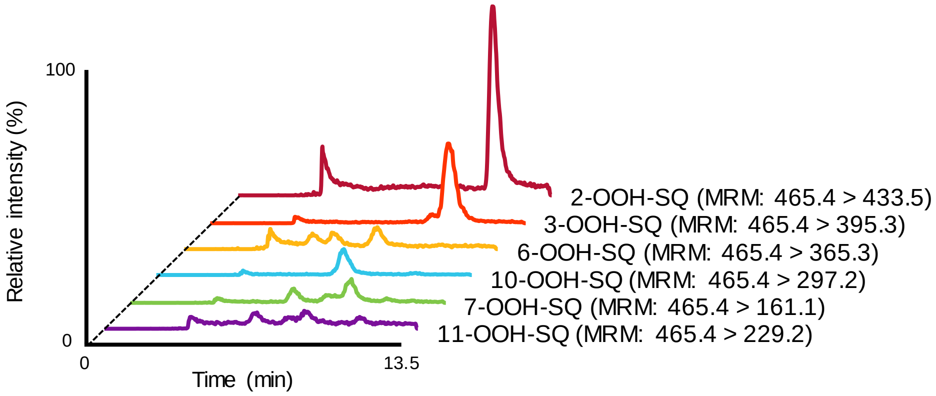

Supplementary Fig. S2. Normal phase LC-MS/MS chromatogram demonstrating the distribution of SQOOH isomers formed by the free radical oxidation of SQ by hydroxyl radicals. In accordance with the free radical oxidation by cumene hydroperoxide, the main SQOOH isomers formed by the oxidation by hydroxyl radicals were 2 and 3-OOH-SQ.

Supplementary Methods

Materials. 2,2'-azobis (4-methoxy-2,4-dimethylvaleronitrile) (MeO-AMVN), $FeSO_4 \cdot 7H_2O$, and 30% H_2O_2 were obtained from Wako Pure Chemical Industries, Ltd. (Osaka, Japan). HEPES was obtained from Sigma-Aldrich (St. Louis, MO, USA). All other materials used are described in the main article.

Free radical oxidation of SQ by MeO-AMVN. In an amber vial, purified SQ (1 mg) was mixed with 10 µL of 0.1 mg/mL MeO-AMVN in chloroform, and the solution was evaporated under nitrogen stream. Oxygen gas was enclosed in the vial, and the cap of the vial was wrapped with Parafilm M (Bermis Company, Inc.; WI, USA) to prevent oxygen from leaking. The sample was heated at 50°C for 2 h, 1 mL of hexane was added, and subjected to normal phase LC-MS/MS analysis as described in the main article.

Free radical oxidation of SQ by hydroxyl radicals. Free radical oxidation of SQ by hydroxyl radicals was performed based on the method by Yoshimura et al.[1] with modifications. To purified SQ (1 mg), 1 mL of distilled water, 50 µL of $FeSO_4 \cdot 7H_2O$ solution (100 mM), 50 µL of HEPES buffer (0.1 M, pH 7.4), and 50 µL of 30% H_2O_2 were added. The solution was incubated at room temperature for 4 h under constant stirring and subjected to extraction by the Folch method[2]. The extracted lipids were dissolved in 1 mL hexane and subjected to normal phase LC-MS/MS analysis as described in the main article.

References

1. Yoshimura, Y. et al. Effects of buffer solutions and chelators on the generation of hydroxyl radical and the lipid peroxidation in the Fenton reaction system. *J. Clin. Biochem. Nutr.* **13**, 147–154 (1992).
2. Folch, J., Lees, M. & Stanley G. H. S. A simple method for the isolation and purification of total lipids from animal tissues. *J. Biol. Chem.* **226**, 497–509 (1957).
